# Premonitory Symptoms of Puerperal Infection

**Published:** 1898-02

**Authors:** 


### PREMONITORY SYMPTOMS OF PUERPERAL INFECTION.

Ferre (*L'Obstetrique*) lays stress on the success of intra-uteri treatment for puerperal fever. The success stands in direct ratio to the earliness of intervention. Hence very careful clinical researches have been made in lying-in hospitals in order to detect true prodromata. The true rigor, local pains and conspicuous pulse and temperature are known to all, and when combined indicate more or less advanced infection. Ferre denies that these symptoms ever come on suddenly, though certain milder types of infection now observed may represent sepsis modified by antiseptic agents. These milder types, however, will assuredly develop into deadly septic infection if neglected. Ferre finds, after long clinical research, that even the severest form is preceded for a day or two by distinct elevation of temperature and pulse, and by insomnia. An evening temperature of about 100 degrees in the axilla, with a fall of about a degree in the morning, without a corresponding drop in a somewhat rapid pulse, is a distinctly suspicious symptom. The rise in the pulse often precedes the rise in the temperature; the observer must therefore make sure that acceleration of the heart's action is accounted for even in a patient who seems otherwise convalescent. Reaction after the fatigue of labor, hemorrhage and emotions all send up the pulse. Insomnia, Ferre has noted, is often observed in the earlier stages of infection, distinct want of sleep without restlessness is usual for a day or two before bad septic symptoms. The lochia may remain free from odor in the premonitory stage of puerperal septicæmia, nor are the discharges always fœtid when the disease is established.—*Canadian Medical Record.*

